# Outcomes of transcatheter aortic valve replacement in younger low-risk patients: a comprehensive meta-analysis of efficacy and safety

**DOI:** 10.3389/fcvm.2025.1586477

**Published:** 2025-08-11

**Authors:** António Rocha de Almeida, Maria Rita Lima, Daniel A. Gomes, Renato Fernandes, Eduardo Infante Oliveira, Pedro Araújo Gonçalves, Rui Campante Teles, Manuel de Sousa Almeida, Lino Patrício

**Affiliations:** ^1^ Unidade Local de Saúde Alentejo Central, Hospital Espírito Santo de Évora, Évora, Portugal; ^2^ Católica Biomedical Research Centre, Faculty of Medicine, Catholic University of Portugal, Oeiras, Portugal; ^3^ Centro de Cardiologia, Hospital Lusíadas Lisboa, Lusíadas Saúde, Lisboa, Portugal; ^4^ Unidade Local de Saúde de Lisboa Ocidental, Hospital de Santa Cruz, Lisbon, Portugal; ^5^ Centro Clinico Academico de Lisboa, Lisboa, Portugal; ^6^ Comprehensive Health Research Center (CHRC), Nova Medical School, Lisbon, Portugal

**Keywords:** TAVI, SAVR, low risk, severe aortic stenosis, younger, short-term

## Abstract

**Background and aims:**

Severe aortic stenosis (AS) was traditionally managed with surgical aortic valve replacement (SAVR). Transcatheter aortic valve implantation (TAVI) emerged as a less invasive alternative, initially for high-risk patients. This meta-analysis evaluates the outcomes of TAVI in younger, low-risk patients, in whom SAVR is currently the gold standard.

**Methods:**

Following PRISMA guidelines, we systematically searched randomized controlled trials (RCTs) comparing TAVI with SAVR in younger (mean age <75 years) low-risk patients (STS score <4%) with severe AS. The primary endpoint was a composite of death or disabling stroke. Secondary endpoints included all-cause mortality, disabling stroke, atrial fibrillation (AF), permanent pacemaker implantation (PPI), bleeding, functional class (NYHA), and quality-of-life (KCCQ score) improvements.

**Results:**

Four RCTs were included with 4,252 patients (2,125 TAVI and 2,127 SAVR). At a mean follow-up of 16 ± 5 months, TAVI showed a non-significant reduction in the composite of death or disabling stroke [2.8% vs. 5.1% risk ratio (RR) 0.98, 95% confidence interval (CI) (0.96–1.00), *p* = 0.11] and all-cause mortality [2.1% vs. 3.7%, RR 0.99, 95% CI (0.97–1.00), *p* = 0.15]. The incidence of disabling stroke was significantly lower in TAVI [0.9 vs. 2.1 RR 0.99, 95% CI (0.98–1.00), *p* < 0.01]. Hospital readmission [7.1% vs. 9.5% RR 0.97, 95% CI (0.96–0.99), *p* < 0.01] and bleeding rates [4.7% vs. 16%, RR 0.87, 95% CI (0.82–0.93), *p* < 0.01] were significantly lower in the TAVI group. Conversely, TAVI had a higher PPI rate [14% vs. 6%, RR 1.08, 95% CI (1.02–1.14), *p* < 0.01]. Faster symptomatic and quality-of-life improvements were sustained in the TAVI group.

**Conclusions:**

TAVI is a viable option for younger low-risk patients with severe AS, being non-inferior to SAVR in short-term outcomes. The benefits of TAVI include a lower risk of disabling stroke, hospital readmission, and bleeding, as well as quicker improvements in symptoms and quality of life. However, higher PPI rates require careful patient selection. The results support a tailored approach to TAVI in younger patients, with ongoing evaluation of long-term outcomes.

**Systematic Review Registration:**

https://www.crd.york.ac.uk/PROSPERO/view/CRD42024559473, PROSPERO (CRD42024559473).

## Introduction

Severe aortic stenosis (AS) is a prevalent condition among the elderly and leads to significant morbidity and mortality (1). Surgical aortic valve replacement (SAVR) has been the gold standard treatment for severe AS (1, 2). However, transcatheter aortic valve implantation (TAVI) has emerged as a minimally invasive alternative, initially reserved for high-risk patients who were considered unsuitable for surgery (3). Over the past decade, the indications for TAVI have increased and include intermediate-risk and, more recently, older low-risk patient populations (1, 2).

The expansion of TAVI into low-risk patients was based on promising initial studies suggesting that TAVI offered similar or superior outcomes than SAVR, with the added benefits of shorter recovery times and reduced perioperative complications (3–6). While recent studies have shown promising results, significant uncertainties persist regarding the efficacy and safety of TAVI in younger low-risk patients (6). In this population, where the well-established gold standard SAVR has known predictable outcomes, the potential complications associated with TAVI and its efficacy become a crucial consideration. Consequently, there remains a need for a comprehensive evaluation of the effectiveness and safety of TAVI in the young, low-risk cohort to support its widespread adoption (3–6).

This systematic review and meta-analysis aims to evaluate the short-term clinical outcomes of TAVI compared with SAVR in younger, low-risk patients. It addresses the uncertainties surrounding the use of TAVI in this specific population, helping to clarify whether TAVI is a viable option for younger, low-risk individuals.

## Methods

### Search of studies and data extraction

We conducted this meta-analysis following the reporting items of the Preferred Reporting Items for Systematic Reviews and Meta-Analysis (PRISMA) recommendations (7). We systematically searched three electronic databases [PubMed, Cochrane Central Register of Controlled Trials (CENTRAL), and Scopus] until June 2024 for all trials comparing TAVI and SAVR in young, low-risk patients with severe AS. No language restrictions were applied. Our search strings included (“severe aortic stenosis” or “severe symptomatic aortic stenosis”), (“TAVI” or “TAVR” or “transcatheter aortic valve replacement” or “transcatheter aortic valve implantation”), (“SAVR” or “surgical aortic valve replacement”), (“low risk”), and (“young”). The authors (AA and ML) independently assessed titles, abstracts, and full texts, when appropriate, for eligibility and data extraction. Disagreements were resolved by consensus, or, if necessary, a third referee would intervene (LP).

Studies were considered eligible if they were (1) randomized controlled trials (RCTs), (2) a comparison of TAVI vs. SAVR in younger (i.e., mean age < 75 years), low-risk patients [i.e., Society of Thoracic Surgeons (STS) 30-day mortality risk score <4%] with severe symptomatic AS (diagnosis according to international recommendations 1, 2), and (3) followed up for at least 1 year. Studies were excluded if they were non-randomized studies without full text published.

**Figure 1:**
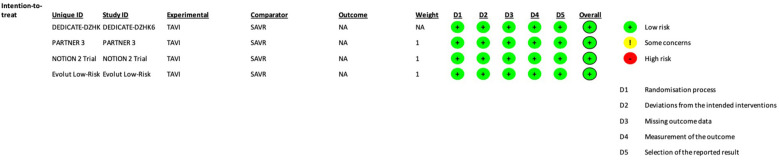
Each trial risk of bias assessment, according to the RoB2 Cochrane tool (8).

The authors extracted data in a previously defined form. The protocol was registered in PROSPERO (CRD42024559473). Two investigators (AA and ML) independently assessed the quality of reporting and risk of bias using the Cochrane Collaboration tool (RoB2) (8). A trial was considered high quality if no domains scored as high risk. The trials were considered to have a low risk of bias, according to RoB2 (8) (Figure 1).

### Clinical endpoint

The primary endpoint evaluated was a composite endpoint of death or disabling stroke, as reported in each trial. Secondary safety endpoints analyzed were all-cause mortality, stroke, hospital readmission, atrial fibrillation (AF), permanent pacemaker implantation (PPI), aortic reintervention, acute kidney injury (AKI), and significant bleeding. Secondary effectiveness endpoints were the New York Heart Association (NYHA) class and the Kansas City Cardiomyopathy Questionnaire (KCCQ) score improvement in both groups. Each trial's definition of each adverse event was used (Supplementary Table 1).

### Statistical analysis

The extracted data were analyzed using the statistical software Review Manager (RevMan) V.5.4.1 (The Cochrane Collaboration) and Stata Statistical Software: Release 18. Risk ratio (RR) and 95% confidence intervals (95% CI) were used as summary statistics to evaluate non-continuous variables, and the Cohen's *d* test for continuous variables. The results were estimated using a random effects model. Non-inferiority was assessed using a predefined margin of 1.05 for the relative risk of death or disabling stroke based on prior evidence and clinical relevance. TAVI was considered non-inferior if the upper bound of the 95% CI for the relative risk did not exceed this margin. Heterogeneity across studies was assessed by *I*^2^ using Cochran's *Q* test, where values of less than 25%, 50%, and 75% were regarded as evidence of low, moderate, and high levels of heterogeneity, respectively. Publication bias was not evaluated as an inadequate number of included trials were used to determine a funnel plot (9).

### Sensitivity analyses

We conducted subgroup analyses to explore heterogeneity among the study population. This subgroup analysis encompassed patients with tricuspid aortic valves and a comparison of trials from the years 2019 and 2024. We conducted the fixed-effect model analysis to verify the consistency of the results.

## Results

### Included studies and patient characteristics

**Figure 2:**
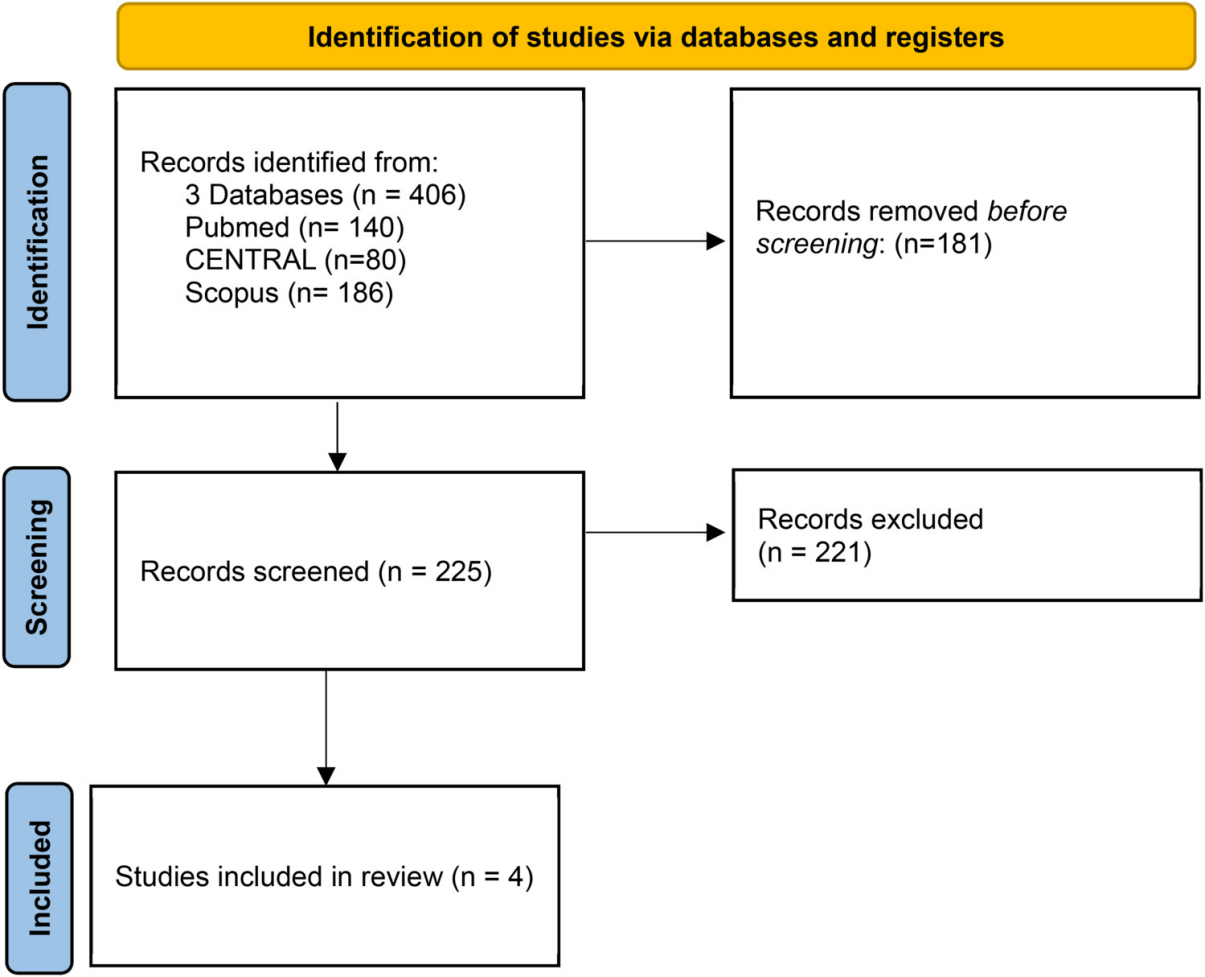
Description of identified studies in databases or registers.

A total of 406 studies were identified through database searching. After removing duplicates, 225 studies were screened. Following the inclusion and exclusion criteria application, four RCTs were included (3–6), involving 4,252 patients: 2,125 (50%) were randomized to TAVI and 2,127 (50%) to SAVR (Figure 2). The weighted mean follow-up duration across all trials was 16 ± 5 months. A list of the patient characteristics of each study is presented in Table 1.

**Table 1:** Study characteristics.

Characteristics	Partner 3	Notion 2	Dedicate DZHK6	Evolut low risk
Year	2019	2024	2024	2019
*N*	1,000	370	1,414	1,468
Mean age (year)	73 ± 6	71 ± 3	74 ± 4	74 ± 6
Sex	31% women	38% women	43% women	35% women
STS score (%)	1.9 ± 0.7	1.1 ± 0.3	1.8 ± 0.1	1.9 ± 0.7
Follow-up	12 months	12 months	12 months	24 months
Inclusion criteria	Severe AS^a^ STS mortality risk < 4% Transfemoral TAVI suitable	Severe AS^a^ ≤75 years of age STS mortality risk <4% Transfemoral TAVI or SAVR suitable	Severe AS^a^ ≥65 years of age Low-intermediate risk score	Severe AS^a^ TAVI or SAVR suitable STS mortality risk <3%
Exclusion criteria	Frailty, bicuspid aortic valve, concomitant severe valvular heart disease	Ascending aorta ≥45 m, CAD not suitable for PCI and CABG.	Significant CAD Previous heart surgery Bicuspid aortic valve Other significant valvular heart disease	Bicuspid aortic valve Concomitant severe valvular heart disease
TAVI type	Balloon-expandable 100%	Self-expandable 73% Balloon-expandable 26%	Self-expandable 35% Balloon-expandable 65%	Self-expandable 100%
Primary outcome	Death, stroke, or rehospitalization	Death, stroke, or rehospitalization	Death or stroke	Death or disabling stroke^b^
Secondary outcomes	Death, death or disabling stroke^b^, stroke, death and stroke, rehospitalization, AF, length of index hospitalization, KCCQ < 45 or KCCQ decrease from baseline ≥10 points, major vascular complications, bleeding complications, acute kidney injury, permanent pacemaker implantation.	Death of any cause, death or disabling stroke^b^, stroke (disabling and non-disabling), major or life-threatening bleeding, new-onset AF, need for PPI, valve endocarditis, valve thrombosis, need for valve re-intervention, and valve performance	Death, Stroke, Stroke or TIA, death or disabling stroke, Disabling stroke^b^, Cardiovascular death, Myocardial infarction, New-onset AF, New-onset LBBB, PPI, Prosthetic valve dysfunction, endocarditis, or thrombosis, Aortic valve reintervention, Major or life-threatening or disabling bleeding, AKI, vascular access-site complication	Death of any cause, death or disabling stroke^b^, Life-threatening bleeding major vascular complications, atrial fibrillation, permanent pacemaker implantation, Stage 2 or 3 acute kidney injury, Prosthetic valve endocarditis, thrombosis, or dysfunction requiring repeat procedure, stroke, and life-threatening bleeding at 12 months

^a^ Severe aortic stenosis defined as aortic valve maximum velocity >4,0 m/s, aortic valvular mean gradient >40 mmHg, or aortic valve area <1.0 cm^2^.

^^b^^ Disabling stroke defined by a score on the modified Rankin scale >2.

AS, aortic stenosis; STS, Society of Thoracic Surgeons; TAVI, transcatheter aortic valve implantation; AF, atrial fibrillation; KCCQ, Kansas City Cardiomyopathy Questionnaire; PPI, permanent pacemaker implantation; PCI, percutaneous coronary intervention; CABG, coronary artery bypass grafting; CAD, coronary artery disease; SAVR, Surgical aortic valve replacement; LBBB, left bundle brunch block; AKI, acute kidney injury. The trial's definition is considered in each outcome.

### Primary outcomes

**Figure 3:**
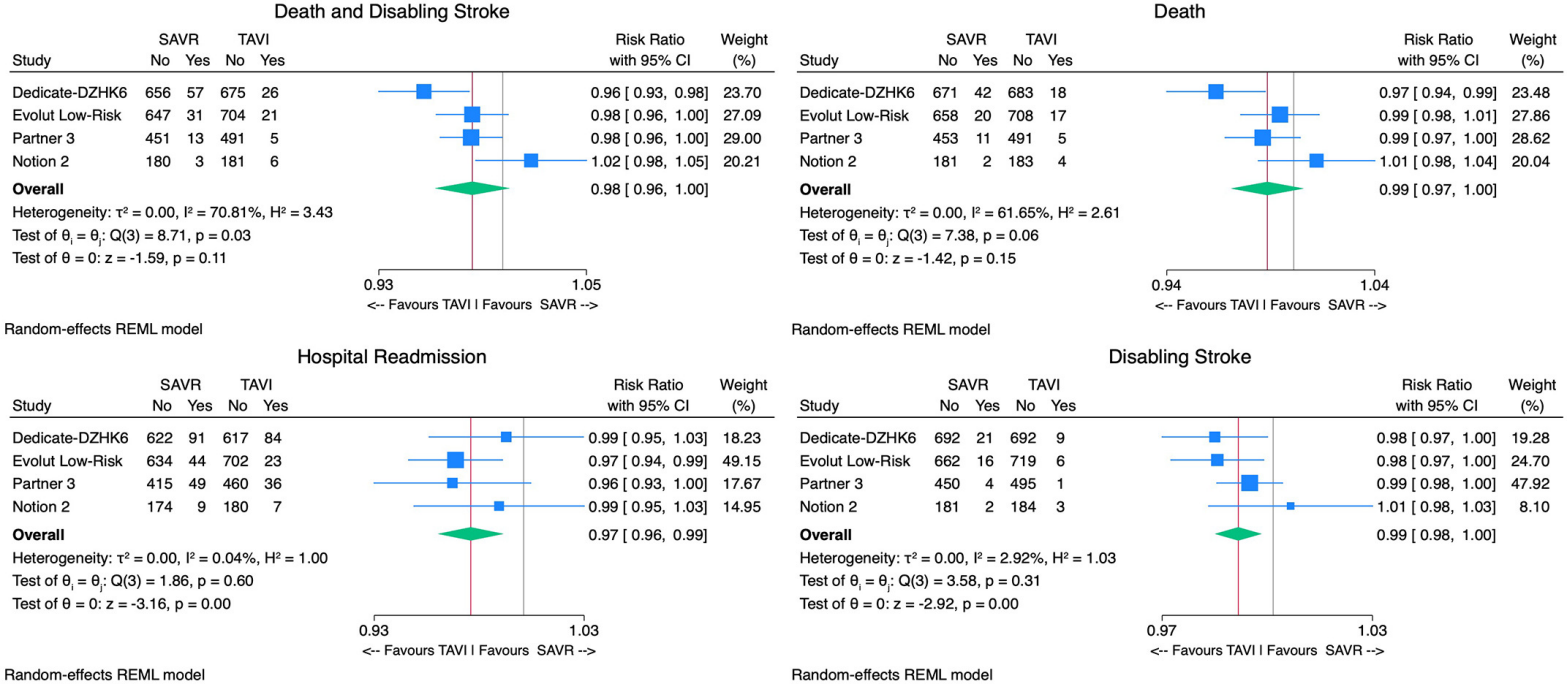
Forest plots comparing the outcome of TAVI and SAVR for the primary endpoint death or disabling stroke, all-cause mortality, disabling stroke, and hospital readmissions.

With regard to the primary outcome, TAVI was associated with a non-significantly lower incidence of death or disabling stroke [2.8% vs. 5.1%, RR 0.98, 95% CI (0.96–1.00), *p* = 0.11, *I*^2^ = 71%] compared with SAVR (Figure 3). The upper bound of the 95% CI (1.00) remained below the predefined non-inferiority margin (1.05), supporting the non-inferiority of TAVI to SAVR for this outcome. The number needed to treat (NNT) in the TAVI arm to prevent one death or disabling stroke was 43 patients.

### Secondary outcomes

Considering safety outcomes, the all-cause mortality rate in the TAVI group was numerically lower than in the SAVR group. However, this difference was not statistically significant [2.1% vs. 3.7%, RR 0.99, 95% CI (0.97–1.00), *p* = 0.15, *I*^2^ = 62%] (Figure 3). On the other hand, the disabling stroke rate was significantly inferior in the TAVI arm [0.9% vs. 2.1% RR 0.99, 95% CI (0.98–0.99), *p* < 0.01, *I*^2^ = 3%] (Figure 3). Non-disabling stroke incidence was similar in both groups [3.1% vs. 3.8% RR 1.00, 95% CI (0.98–1.02), *p* = 0.67, *I*^2^ = 66%]. TAVI's cohort rehospitalization incidence was significantly lower than SAVR's [7.1% vs. 9.5% RR 0.97, 95% CI (0.96–0.99), *p* < 0.01, *I*^2^ = 0%] (Figure 3).

**Figure 4:**
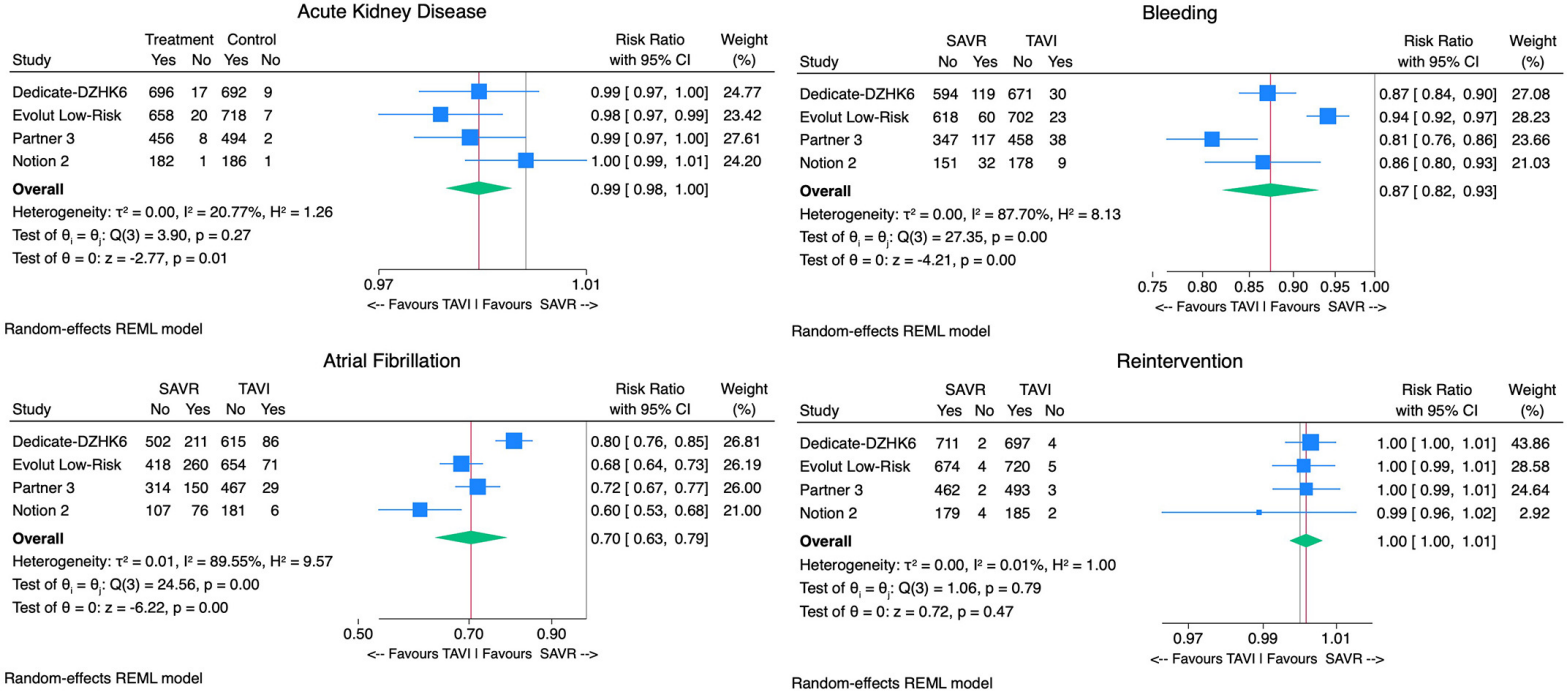
Forest plots comparing the outcome of TAVI and SAVR postprocedural atrial fibrillation, aortic reintervention, acute kidney injury, and bleeding.

**Figure 5:**
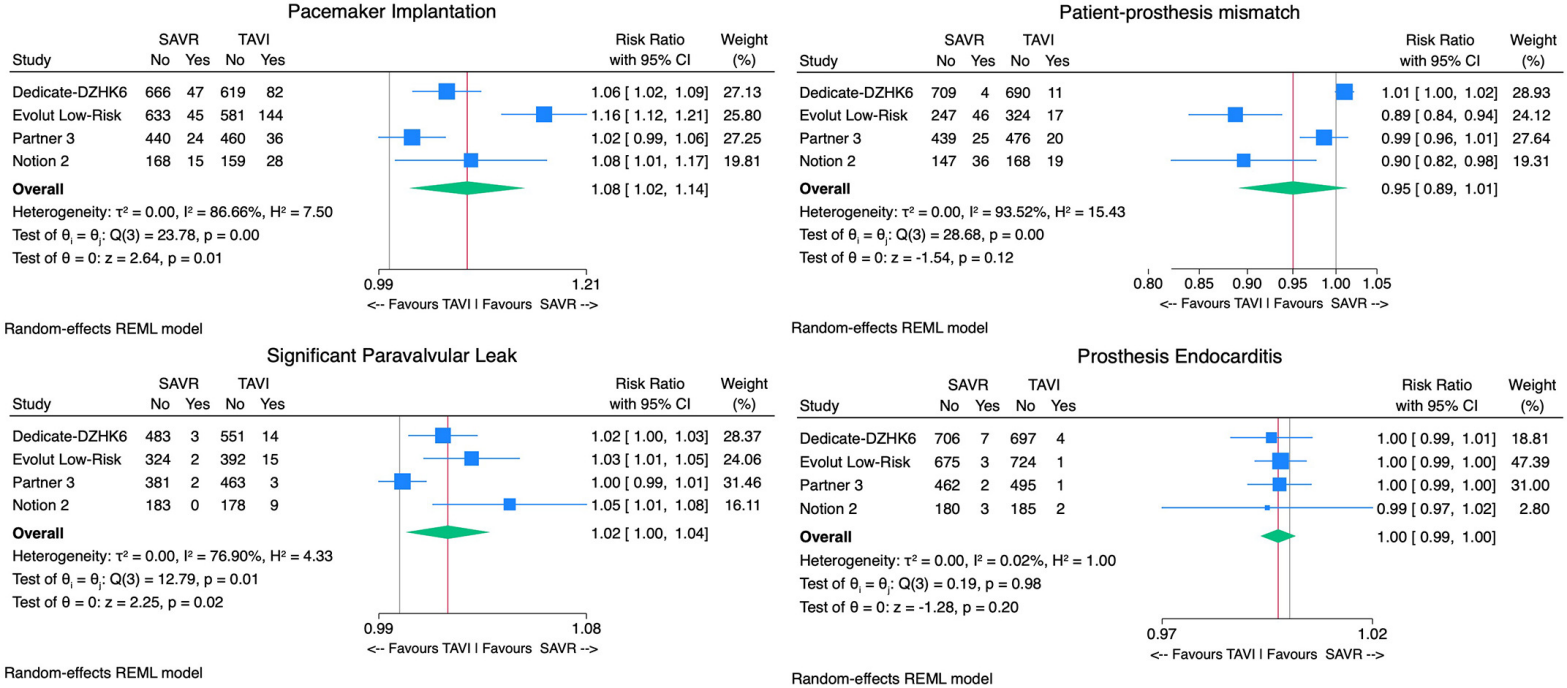
Forest plots comparing the outcome of TAVI and SAVR permanent pacemaker implantation, patient-prosthesis mismatch, endocarditis, and significant paravalvular leak.

The TAVI group demonstrated significantly lower rates of postprocedural AF [9% vs. 34% RR 0.70, 95% CI (0.63–0.79), *p* < 0.01, *I*^2^ = 90%], AKI [0.9% vs. 2.2% RR 0.99, 95% CI (0.98 −1.00), *p* < 0.01, *I*^2^ = 21%], and bleeding rates when compared with SAVR [4.7% vs. 16.1% RR 0.87, 95% CI (0.82–0.93), *p* < 0.01, *I*^2^ = 88%]. However, TAVI was associated with higher PPI [13.7% vs. 6.4% RR 1.08, 95% CI (1.02; 1.14), *p* < 0.01, *I*^2^ = 87%]. No significant difference in aortic valve reintervention between groups was found [0.7% vs. 0.6% RR 1.00 95%CI (1.00–1.01), *p* = 0.8, *I*^2^ = 0%] (Figure 4). With regard to prosthesis-related outcomes, the TAVI group experienced higher rates of significant paravalvular leak (PVL) [2.5% vs. 0.5% RR 1.02, 95% CI (1.00–1.04), *p* < 0.01, *I*^2^ = 77%]. There were no statistically significant differences in prosthesis thrombosis [0.6% vs. 0.3% RR 1.00, 95% CI (1.00–1.01), *p* = 0.4, *I*^2^ = 18%], endocarditis [0.4% vs. 0.7% RR 1.00, 95% CI (0.99–1.00), *p* = 0.2, *I*^2^ = 0%], and patient-prosthesis mismatch (PPM) [3.2% vs. 5.4% RR 0.95, 95% CI (0.89–1.01), *p* = 0.16, *I*^2^ = 89%] between groups (Figure 5). The mean gradient across the aortic prosthesis was slightly lower in the TAVI group (10.8 mmHg vs. 11.4 mmHg), although the difference was not statistically significant (Cohen's *d* 0.40, *p* = 0.8, *I*^2^ = 99%). Similarly, no significant difference was observed in the aortic prosthesis valve area, with TAVI showing an AVA of 1.80 cm^2^ compared with 1.75 cm^2^ for SAVR (Cohen's *d* = −0.64, *p* = 0.7, *I*^2^ = 100%) (Supplementary Figure 1).

The TAVI group exhibited greater improvement in KCCQ scores at 1 month [19% vs. 6.3%; Cohen's *d* 12.1, 95% CI (6.2–18.2), *p* < 0.01, *I*^2^ = 99%]. However, no significant differences were observed between the groups at 1 year (19.9% vs. 18.8%; Cohen's *d* 0.74, *p* = 0.3, *I*^2^ = 99%). Similar results were found for NYHA improvement. TAVI was linked to a shorter hospital stay, with an average of 4 days compared with 8 days [Cohen's *d* 0.48, 95% CI (0.23–0.98)] (Supplementary Figures 2 and 3).

### Subgroup and sensitivity outcomes

**Figure 6:**
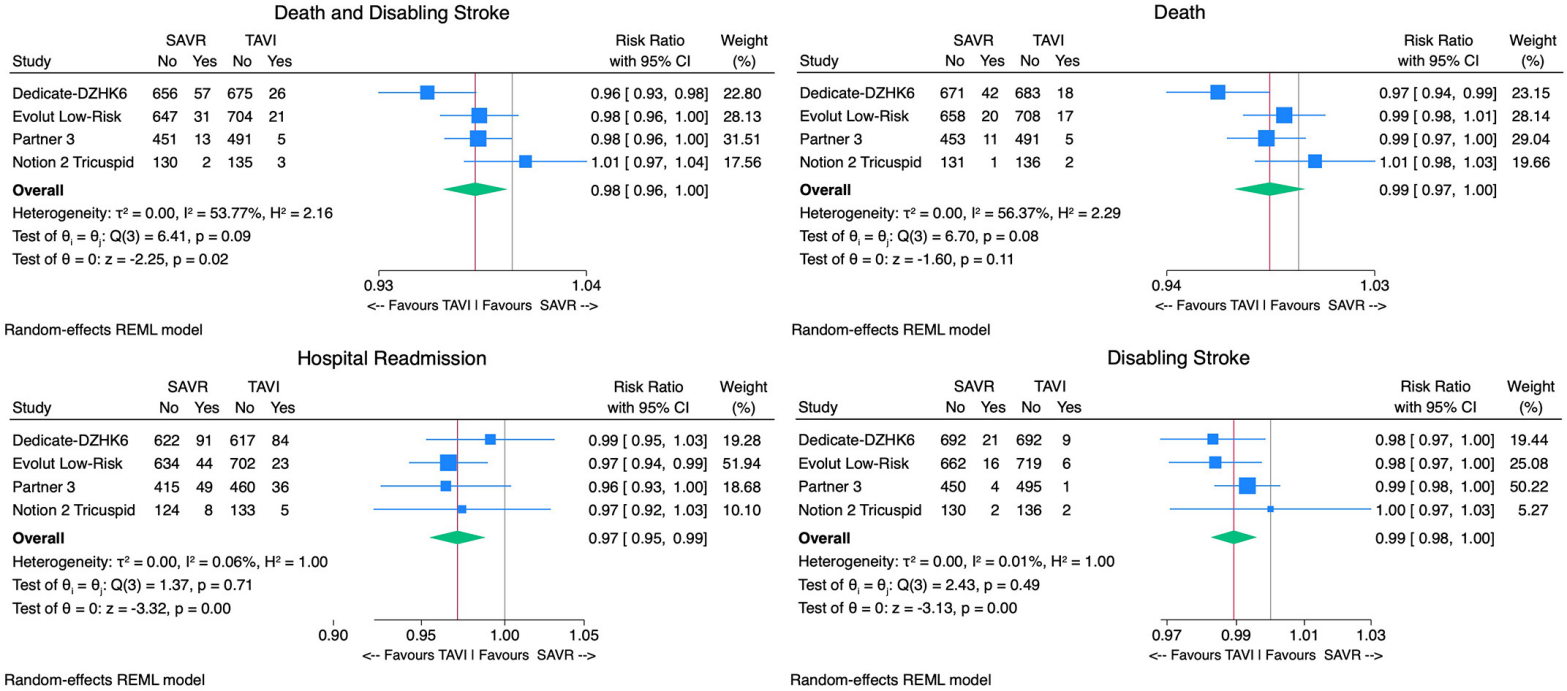
Forest plots of subgroup analysis comparing the outcome of TAVI and SAVR in tricuspid aortic valve patients for the primary endpoint death or disabling stroke, all-cause mortality, disabling stroke, and hospital readmissions.

The subgroup of patients with tricuspid aortic valves included 4,047 patients: 2,060 underwent TAVI, and 1,987 received SAVR. TAVI was associated with a statistically significant reduction in the primary outcome of death or disabling stroke compared with SAVR [2.7% vs. 5.2% RR 0.98, 95% CI (0.96–1.00), *p* = 0.02], with moderate heterogeneity (*I*^2^ = 54%). While TAVI also showed a trend toward reduced mortality, this was not statistically significant [2.1% vs. 3.7%; RR 0.99, 95% CI (0.97–1.00), *p* = 0.1]. In addition, TAVI showed a significant reduction in disabling stroke [0.9% vs. 2.2%; RR 0.99, 95% CI (0.98–1.00), *p* < 0.01] (Figure 6).

To evaluate the impact of publication year on outcomes, we focused on trials published in 2019 (Partner 3 and Evolut Low Risk) vs. those published in 2024 (Dedicate-DZHK6 and Notion 2). The 2019 trials demonstrated a statistically significant reduction in the primary outcome of death or disabling stroke, favoring TAVI [RR 0.98, 95% CI (0.97–0.99), *p* = 0.01] with negligible heterogeneity (*I*^2^ = 0%). Conversely, the 2024 trials showed no significant difference between TAVI and SAVR [RR 0.98, 95% CI (0.92–1.05), *p* = 0.62], with high heterogeneity (*I*^2^ = 89%). Despite these differences, the overall test for subgroup differences was not statistically significant (*p* = 0.93), suggesting no evident effect modification based on the publication year (Supplementary Figure 4).

The fixed-effects model analysis demonstrated a statistically significant reduction in the primary outcome of death or disabling stroke [RR 0.98 (95% CI 0.96–0.99), *p* < 0.01, *I*^2^ = 58%], resembling the random-effects model, which showed a reduction, yet not statistically significant. For disabling stroke, the fixed-effects model indicated a reduction of the outcome [RR 0.99 95% CI (0.98–0.99), *p* < 0.01, *I*^2^ = 22%], while for death individually**,** a similar statistically significant reduction was observed [RR 0.98 95% CI (0.97–0.99), *p* = 0.03, *I*^2^ = 62%]. This aligns with the trends observed in the random-effects models, providing further support for the consistency of these results (Supplementary Figures 5–7).

## Discussion

This meta-analysis comparing TAVI and SAVR in younger, low-risk patients yields pivotal insights, contributing to the evolving paradigm of aortic valve replacement strategies. The pooled data included a cohort of 4,252 participants, and the outcomes of the study provide a nuanced understanding of the short-term efficacy and safety profiles of these interventions.

The primary outcome of death or disabling stroke occurred numerically at a lower rate in the TAVI group compared with the SAVR group (2.8% vs. 5.1%). However, this difference was not statistically significant (*p* = 0.11). This finding supports the non-inferiority of TAVI, indicating that TAVI might be an alternative option to SAVR, the traditional gold standard for this population (10–12). In addition, TAVI showed a significantly lower incidence of disabling stroke (0.9% vs. 2.1%, *p* < 0.01), highlighting its superior safety profile compared with SAVR for this crucial outcome. While the reduction in all-cause mortality was not statistically significant, it reinforces TAVI as a safe and viable alternative to SAVR. The minimally invasive nature of TAVI, coupled with advances in procedural techniques and valve development, likely contributes to these favorable outcomes (13, 14).

With regard to secondary endpoints, hospital readmissions were significantly lower in the TAVI group than in the SAVR group (7.1% vs. 9.5%, *p* < 0.01). Reduced readmission rates translate to lower healthcare costs and better quality of life for patients (15). Furthermore, TAVI was associated with substantially lower rates of postprocedural AF (9% vs. 34%, *p* < 0.01) and AKI (0.9% vs. 2.2%, *p* < 0.01), which may be attributed to its less invasive, lighter procedural burden and reduced systemic impact compared with SAVR. Postprocedural AF's long-term impact remains unclear, yet it may increase the risk of future AF, potentially necessitating anticoagulation and influencing long-term outcomes (16). Nonetheless, TAVI demonstrated a significantly higher incidence of PPI (13.7% vs. 6.4%, *p* < 0.01). The PPI rates are similar to those in intermediate-to-high-risk patient trials (17, 18). The higher PPI rates in TAVI remain a relevant consideration in younger patients, emphasizing careful patient selection and management, although prior evidence shows no effect on survival (19).

In addition, the lower bleeding rate in TAVI (4.7% vs. 15.7%, *p* < 0.01) is a significant advantage, as bleeding can lead to prolonged hospital stays and other complications such as anemia and a slower recovery time. This finding further reinforces the minimally invasive nature of TAVI as beneficial in reducing perioperative risks.

While TAVI shows a higher rate of PVL (2.5% vs. 0.5%, *p* < 0.01), other prosthesis-related outcomes, such as thrombosis and endocarditis, are similar to SAVR. Preprocedural imaging and optimization techniques are key to optimizing outcome results. Despite the higher PVL rate, TAVI remains a viable option with comparable outcomes to SAVR, and ongoing advancements are expected to address current limitations. As measured by the KCCQ, patients undergoing TAVI reported better quality of life at 1 month than SAVR (19.1% vs. 6.3%, *p* < 0.01), suggesting faster recovery and superior early postprocedural outcomes. Similarly, NYHA functional class improvements were more pronounced in the TAVI group during the early follow-up. At 1 year, these differences became similar between groups, indicating that the early benefits of TAVI were maintained. The significantly shorter hospital stays for TAVI patients (4 days vs. 8 days) further support its role in improving early recovery and quality of life and reducing healthcare resource.

A significant aspect of this meta-analysis is the variable heterogeneity observed across the outcomes. Three of the four trials excluded patients with bicuspid aortic valves—the NOTION2 trial included a significant proportion of patients with bicuspid aortic valves (26%). The subgroup analysis focused exclusively on patients with tricuspid aortic valves demonstrated a lower incidence of the primary outcome of death or disabling stroke in the TAVI cohort compared with SAVR (2.7% vs. 5.2%, *p* = 0.02), reinforcing its safety within this subgroup. Moreover, the heterogeneity for this outcome was moderate (*I*^2^ = 54%), suggesting consistent findings across studies. When analyzing the components of the primary outcome, TAVI exhibited a similar all-cause mortality rate compared with SAVR (2.1% vs. 3.7%, *p* = 0.08). For disabling stroke, TAVI demonstrated a significant reduction compared with SAVR (0.9% vs. 2.2%, *p* < 0.01). This finding demonstrates the safety profile of TAVI regarding major cerebrovascular complications, as in the general population, making it an appealing option for patients with tricuspid aortic valves.

Notably, several secondary outcomes, particularly postprocedural AF, bleeding, and PPI, exhibited high heterogeneity, which might be associated with potential contributors, such as differences in valve type (self-expanding vs. balloon-expandable), operator experience, postprocedural management, and patient selection criteria. In addition, these multicenter trials were conducted in varying geographical contexts, with some predominantly enrolling patients in the United States and others across European centers. This might have introduced variability related to regional practice, procedural protocols, and postprocedural care strategies. Recognizing these factors is essential when interpreting and applying these findings in clinical practice. In addition, prosthesis type (self-expanding vs. balloon-expandable) may have contributed to heterogeneity in several outcomes. While some trials used a single valve platform, others included both types of devices in varying proportions (Table 1). Unfortunately, detailed outcome data by prosthesis type were not consistently reported across studies, limiting the feasibility of a dedicated subgroup analysis. Nevertheless, this difference in device selection may account for part of the observed heterogeneity and should be considered when interpreting procedural and clinical endpoints across studies.

Overall, the subgroup analysis emphasizes the advantages of TAVI in reducing the primary composite endpoint and disabling stroke, while maintaining non-inferiority in survival outcomes compared with SAVR. These results support the consideration of TAVI as a viable alternative to SAVR in cases of severe tricuspid AS.

In addition, analysis by publication year indicated that the benefits of TAVI over SAVR have remained consistent in recent years, despite earlier trials showing statistically significant reductions in the primary endpoint, while more recent trials did not. The difference between the 2019 and 2024 trials was not statistically significant. One factor that may have contributed to the lack of significant difference in the 2024 trials was the inclusion of bicuspid patients in one of the studies (NOTION-2 trial).

A key consideration in this younger, low-risk population is the role of a dedicated multidisciplinary Heart Team in the decision-making process. Treatment selection should account for immediate procedural outcomes and long-term management, patient preferences, comorbidities, and anatomical factors. The role of the Heart Team is to take a comprehensive approach, ensuring appropriate treatment choice, while also formulating a tailored lifetime management plan, which is essential, as younger patients are more likely to require future interventions. Given their longer life expectancy, concerns such as structural valve deterioration, durability of bioprostheses, and the likelihood of requiring repeat interventions (including valve-in-valve procedures) or preservation of coronary access become especially pertinent. These considerations are critical in the decision-making process and highlight the need for long-term data and carefully designed management strategies tailored to this younger cohort. Careful procedural planning should therefore consider prosthesis type, implantation depth, and coronary anatomy to optimize long-term treatment options.

The evidence from this meta-analysis supports TAVI as a safe, effective, and less invasive technique for younger, low-risk patients with severe tricuspid AS. The advantages of reduced disabling strokes, rehospitalization, and perioperative complications, coupled with improved early recovery and quality of life, support the broader use of TAVI in this population. However, its limitations, particularly concerning PPI and paravalvular leaks, warrant refinement of ongoing device design and procedural techniques.

### Limitations

Although the trials focused on a younger, low-risk population, the mean age of participants ranged from 71 ± 3 to 74 ± 6 years. This indicates that in most studies, approximately half of the participants were over 75, potentially influencing the overall outcomes. The NOTION 2 trial was an exception, including patients younger than 75. Subgroup analyses consistently showed similar results for participants under 75 years across trials. However, the endpoints assessed in these subgroup analyses varied, limiting a comprehensive evaluation for this younger cohort. While younger patients are presumed to maintain the benefits, further studies focusing on young individuals, under 75, are essential to provide clearer insights into this group.

This meta-analysis primarily focuses on short-term outcomes; however, long-term results are essential in this population. With an average follow-up of 16 ± 5 months, it is insufficient to draw definitive conclusions regarding valve durability, structural deterioration, coronary access, and the feasibility of valve-in-valve procedures over time. Since TAVI has primarily been used in older or higher-risk patients with shorter life expectancies, longer follow-up studies are crucial to assess valve durability and structural deterioration. These issues are particularly relevant in younger patients with a longer life expectancy and an increased likelihood of requiring repeat interventions or future coronary procedures. While short-term data confirm that TAVI is a safe and effective alternative, comprehensive long-term evidence is needed to define its role in lifetime management strategies fully.

Furthermore, due to the absence of specific data, a sensitivity analysis comparing self-expanding and balloon-expandable prostheses results could not be conducted, which limits insights into how prosthesis type might influence clinical outcomes for this population. In addition, some heterogeneity exists in the trials, as some utilized exclusively balloon-expandable or self-expanding valves, while two studies included a mixed proportion of both (Table 1).

## Conclusion

This meta-analysis provides evidence favoring TAVI in low-risk younger patients with severe AS exhibiting non-inferiority to SAVR regarding periprocedural outcomes and early follow-up. The similar primary composite endpoint of all-cause mortality or disabling stroke rate, along with lower disabling stroke rates, underscores the efficacy and safety of TAVI, confirming it as a viable alternative to SAVR in this population. These benefits are more pronounced in patients with tricuspid aortic valves, while uncertainties regarding TAVI persist in bicuspid aortic valve cases. The higher PPI and paravalvular leak rates in the TAVI group highlight the need for careful patient selection.

These findings reflect the evolving paradigm of aortic valve interventions. TAVI should no longer be reserved exclusively for older or higher-risk patients but should be considered within an individualized, Heart Team–led strategy that accounts for multiple patient-specific factors. As its use extends to younger populations, long-term data on valve durability, reinterventions, and coronary access will be essential to define its role in lifetime management fully.

## Data Availability

The raw data supporting the conclusions of this article will be made available by the authors, without undue reservation.
