# Supplementary material for: Outcomes of transcatheter aortic valve replacement in younger low-risk patients: a comprehensive meta-analysis of efficacy and safety
Source: Front Cardiovasc Med. 2025 Aug 11;12:1586477. doi: 10.3389/fcvm.2025.1586477 (PMC12375568; doi:10.3389/fcvm.2025.1586477)
Supplement: Supplementary file 1 [file Datasheet1.pdf]

Supplementary Material

Section A: Trial's outcome definition

Outcome	
**Partner 3**	
Disabling stroke	mRS ≥ 2 or more at 90 days (or last clinical visit) and an increase of at least one mRS category from an individual's pre-stroke baseline
Hospital readmission	Any admission ≥ 24h to the hospital for either a diagnostic or therapeutic purpose following discharge from the index hospitalization, valve-related, procedure related or heart failure
Post-procedural Atrial fibrillation	New onset clinical atrial fibrillation at 30 days. New-onset atrial fibrillation (or flutter) is diagnosed if it is recorded on a 12-lead ECG, or at least 30 seconds on a rhythm strip
Acute kidney injury	Abrupt loss of kidney function, resulting in the retention of urea and other nitrogenous waste products:<br>Stage 2: Increase in sCreatinine 2-3x or Urine output <0.5 ml/kg per hour for >12 but <24 hours<br>Stage 3 Increase in sCreatinine to ≥3% or serum creatinine of ≥4.0 mg/dL (≥354 mmol/L) with an acute increase of at least 0.5 mg/dL (44 mmol/L) or Urine output <0.3 ml/kg per hour for ≥24 hours or Anuria for ≥12 hours<br>Patients receiving renal replacement therapy (dialysis, hemodialysis, peritoneal dialysis, hemofiltration, transplant) are considered to meet Stage 3 criteria irrespective of other criteria
Reintervention	Any operation that repairs, alters, or replaces a previously operated valve and refers to events occurring for any reason after the index procedure. These interventions include:<br>- Balloon aortic valvuloplasty<br>- Surgical aortic valve replacement<br>- Valve in valve<br>- Percutaneous paravalvular leak closure
Prosthesis-patient mismatch	Moderate: Indexed<br>EOA 0.85–0.65 cm2/m2 (0.70–0.55 cm2/m2 if BMI < 30kg/m2)<br>Severe: Indexed EOA<br><0.65 cm2/m2 (<0.55 cm2/m2 if BMI > 30 kg/m2)

Bleeding	Severity:<br>Life-threatening or disabling bleeding<br>• Fatal bleeding OR<br>• Bleeding in a critical organ, such as intracranial, intraspinal,<br>intraocular, or pericardial necessitating pericardiocentesis, or intramuscular with compartment syndrome) OR<br>• Bleeding causing hypovolemic shock or severe hypotension requiring vasopressors or surgery OR<br>• Overt source of bleeding with drop in hemoglobin ≥5 g/dL or whole blood or packed red blood cells transfusion ≥ 4 units<br>Major bleeding:<br>• Overt bleeding either associated with a drop in the hemoglobin level of at least 3.0 g/dL or requiring transfusion of two or three units of whole blood/RBC, or causing hospitalization or permanent injury, or requiring surgery AND<br>• Does not meet criteria of life-threatening or disabling bleeding
Endocarditis	Fulfillment of the Duke endocarditis criteria OR Abscess, paravalvular leak, pus, or vegetation secondary to infection by histological or bacteriological studies during a re-operation OR Findings of abscess, pus, or vegetation involving a repaired or replaced valve during an autopsy
**Evolut low-risk**	
Disabling stroke	mRS score of ≥ 2 at 90 days and an increase in at least 1 mRS category from an individual's pre-stroke baseline
Acute kidney injury	Stage 2<br>1) Increase in serum creatinine to 200 – 299% (2.0-2.99 times increase compared with baseline) OR<br>2) 2) Urine output <0.5 mL/kg/h for >12 but <24 h<br>Stage 3<br>1) Increase in serum creatinine to ≥ 300% (3 times increase compared with baseline) OR serum<br>creatinine of ≥4.0 mg/dL (≥354 mmol/L) with an acute increase of at least 0.5 mg/dL (44 mmol/L)<br>OR<br>2) Urine output <0.3 ml/kg/h for ≥24 h OR<br>3) Anuria for ≥12 h
Bleeding	Life-threatening or disabling bleeding<br>1) Fatal bleeding (Bleeding Academic Research Consortium [BARC] type 5) OR<br>2) Bleeding in a critical organ, such as intracranial, intraspinal, intraocular, or pericardial

	necessitating pericardiocentesis, or intramuscular with compartment syndrome (BARC type 3b<br>and 3c) OR<br>3) Bleeding causing hypovolemic shock or severe hypotension requiring vasopressors or surgery<br>(BARC type 3b) OR<br>4) Overt source of bleeding with drop in hemoglobin ≥5 g/dL or whole blood or packed red blood cells (RBCs) transfusion ≥4 units* (BARC type 3b)<br>Major bleeding<br>1) Overt bleeding either associated with a drop in the hemoglobin level of at least 3.0 g/dL or<br>requiring transfusion of 2 or 3 units of whole blood/RBC, or causing hospitalization or permanent injury, or requiring surgery AND<br>2) Does not meet criteria of life-threatening or disabling bleeding
Endocarditis	Fulfillment of the Duke endocarditis criteria OR Abscess, paravalvular leak, pus, or vegetation secondary to infection by histological or bacteriological studies during a re-operation OR Findings of abscess, pus, or vegetation involving a repaired or replaced valve during an autopsy
**Notion 2**	
Disabling stroke	mRS score of 2 or more at 90 days and an increase in at least one modified RS category from an individual's pre-stroke baseline
Rehospitalization	Any admission to the hospital for either a diagnostic or therapeutic purpose following discharge from the index hospitalization, due to valve-related, procedure-related rehospitalization, heart failure related hospitalization. It includes either:<br>- Admission to an inpatient unit (treated by a physician in a hospital for at least a 24hour period), OR<br>- Visit to an Emergency Room/Observation unit longer than 24 hours
Acute Kidney injury	Stage 2 -Increase in serum creatinine >2–3x within 7 days compared with baseline<br>Stage 3 - fulfils at least one of the following criteria:<br>- Increase in serum creatinine >300% (>3.0x increase) within 7 days compared with baseline<br>- Serum creatinine > 354 μmol/L with an acute increase of > 44 μmol/L<br>Stage 4 - Requiring new temporary or permanent renal replacement therapy

Prosthesis-patient mismatch	Indexed estimated orifice area (iEOA) cm2/m2<br>In case of BMI < 30 kg/m2<br>• Moderate: 0.85 – 0.66<br>• Severe: < 0.65<br>In case of BMI > 30 kg/m2<br>• Moderate: 0.70 – 0.56<br>• Severe: < 0.55
Endocarditis	Fulfillment of the Duke endocarditis criteria OR Abscess, paravalvular leak, pus, or vegetation secondary to infection by histological or bacteriological studies during a re-operation OR Findings of abscess, pus, or vegetation involving a repaired or replaced valve during an autopsy
**Dedicate-DZHK6**	
Disabling stroke	a modified Ranking Scale (mRS) score of 2 or more at 90 days and an increase in at least one mRS category from an individual's pre-stroke baseline
Rehospitalization	Any admission to the hospital for either a diagnostic or therapeutic purpose following discharge from the index hospitalization, due to valve-related, procedure-related rehospitalization, heart failure related hospitalization. It includes either:<br>- Admission to an inpatient unit (treated by a physician in a hospital for at least a 24hour period), OR<br>- Visit to an Emergency Room/Observation unit longer than 24 hours
Acute Kidney injury	Stage 2 -Increase in serum creatinine >2–3x within 7 days compared with baseline<br>Stage 3 - fulfils at least one of the following criteria:<br>- Increase in serum creatinine >300% (>3.0x increase) within 7 days compared with baseline<br>- Serum creatinine > 354 µmol/L with an acute increase of > 44 µmol/L<br>Stage 4 - Requiring new temporary or permanent renal replacement therapy
Bleeding	Life-threatening or disabling bleeding<br>Fatal bleeding (Bleeding Academic Research Consortium2 [BARC] type 5) OR Bleeding in a critical organ, such as intracranial, intraspinal, intraocular, or pericardial necessitating pericardiocentesis, or intramuscular with compartment syndrome (BARC 2 type 3b and 3c) OR Bleeding causing hypovolaemic shock or severe hypotension requiring vasopressors or surgery (BARC 2 type 3b) OR Overt source of bleeding with drop in haemoglobin >5 g/dL or whole blood or packed red blood cells (RBCs) transfusion >4 units (BARC 2 type 3b)

	Major bleeding (BARC 2 type 3a)<br>Overt bleeding either associated with a drop in the hemoglobin level of at least 3.0 g/dl or requiring transfusion of two or three units of whole blood/RBC, or causing hospitalization or permanent injury, or requiring surgery AND does not meet criteria of life-threatening or disabling bleeding
Prosthesis-patient mismatch	Indexed estimated orifice area (iEOA) cm2/m2<br>In case of BMI < 30 kg/m2<br>• Moderate: 0.85 – 0.66<br>• Severe: < 0.65<br>In case of BMI > 30 kg/m2<br>• Moderate: 0.70 – 0.56<br>• Severe: < 0.55
Endocarditis	Fulfillment of the Duke endocarditis criteria OR Abscess, paravalvular leak, pus, or vegetation secondary to infection by histological or bacteriological studies during a re-operation OR Findings of abscess, pus, or vegetation involving a repaired or replaced valve during an autopsy

Table 1. Outcomes definition by each trial. BMI – body mass index; EOA – estimated orifice area; mRS – modified Rankin Scale,

Section B Prosthesis outcome forrest plots

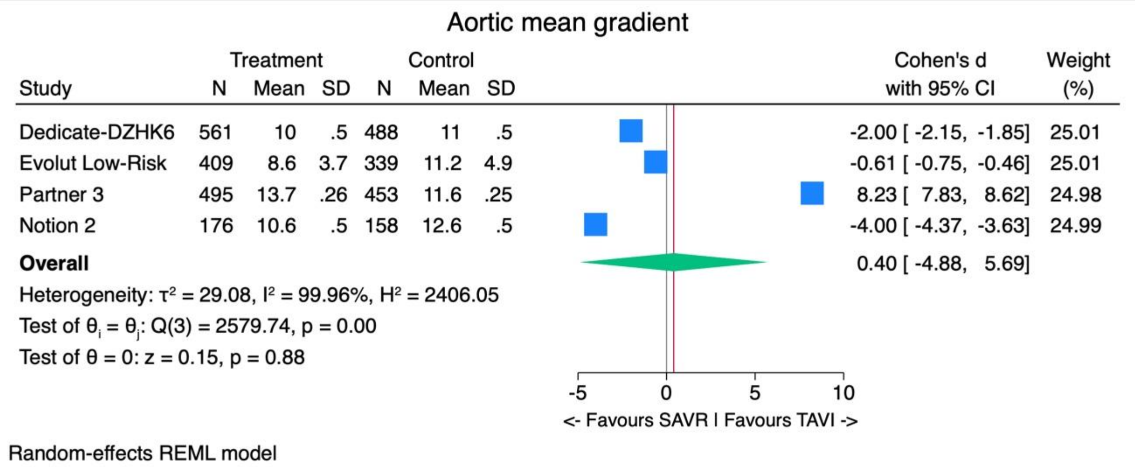

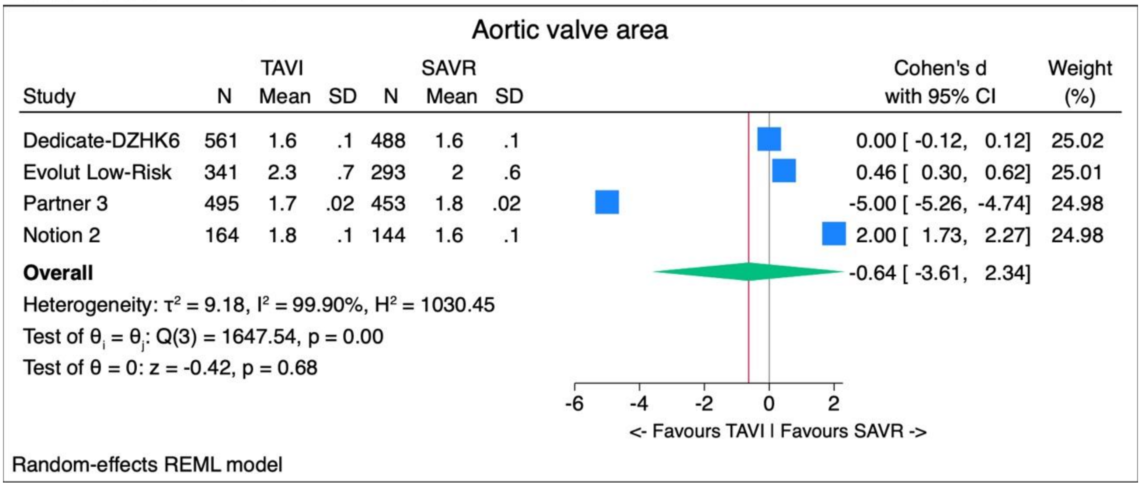

Section C Functional Class NYHA II-IV after 1 month and 1 year

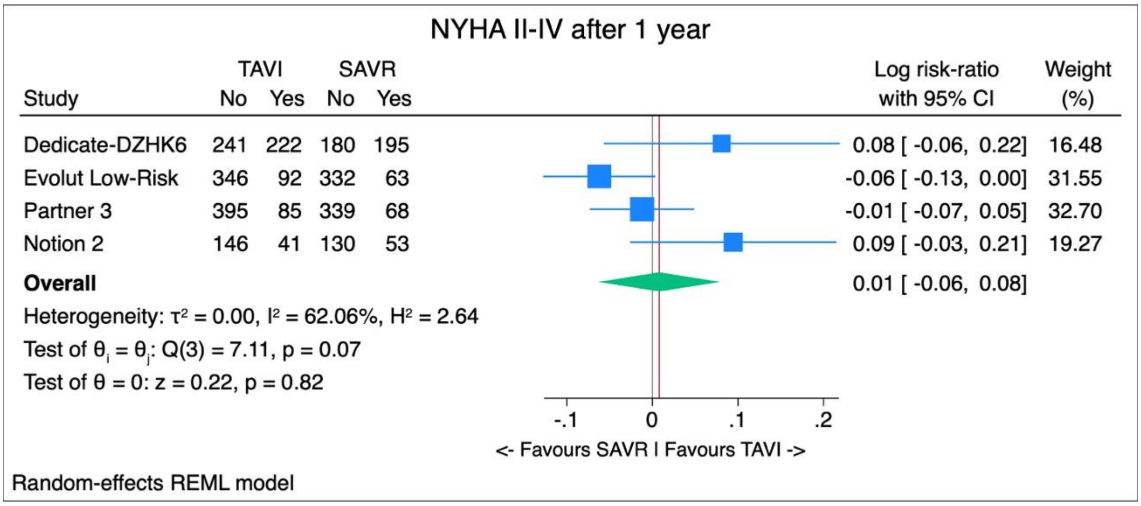

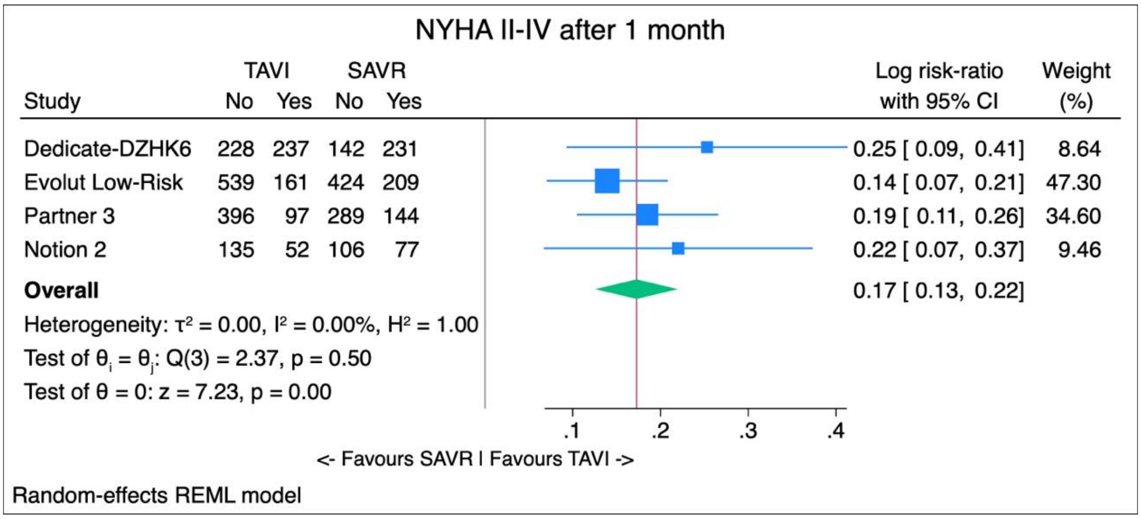

Section D Quality of life mean improvement after 1 month and 1 year according to KQQC mean value.

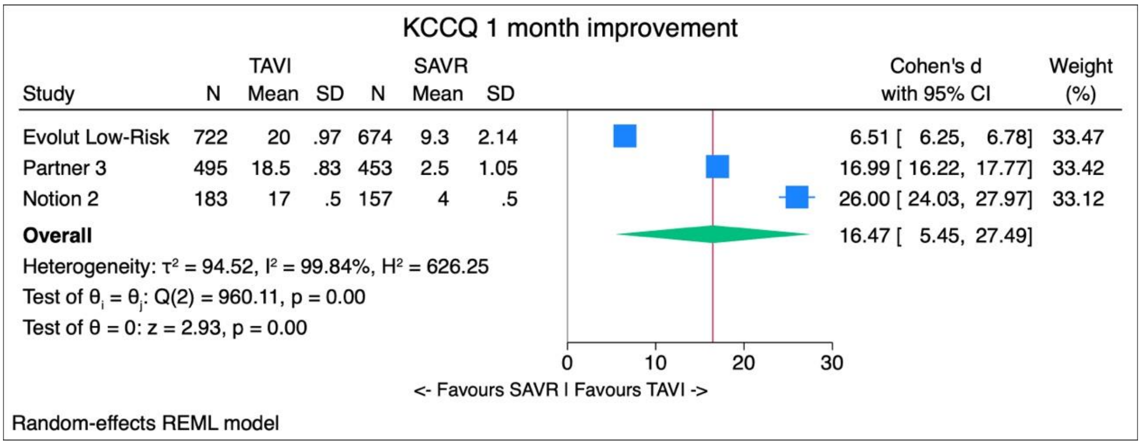

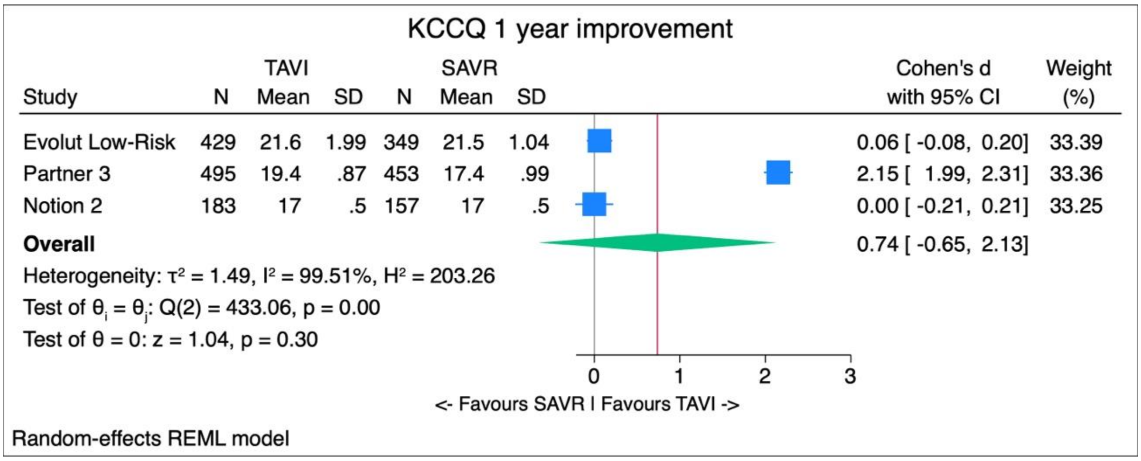

Section E Subgroup analysis of death or disabling stroke by trial's publication year

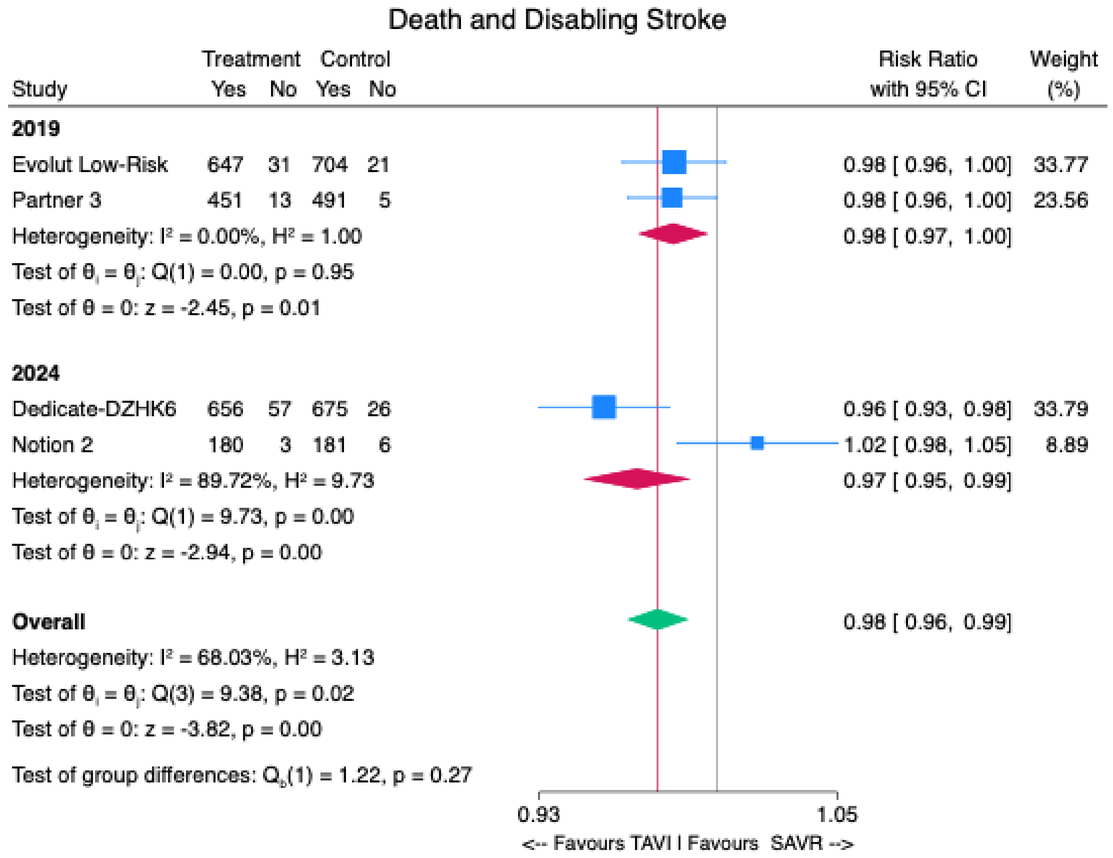

Fixed-effects Mantel–Haenszel model

Section F: Fixed event model analysis of the primary outcome of composite death or disabling stroke, death and disabling stroke outcomes

Death and Disabling Stroke

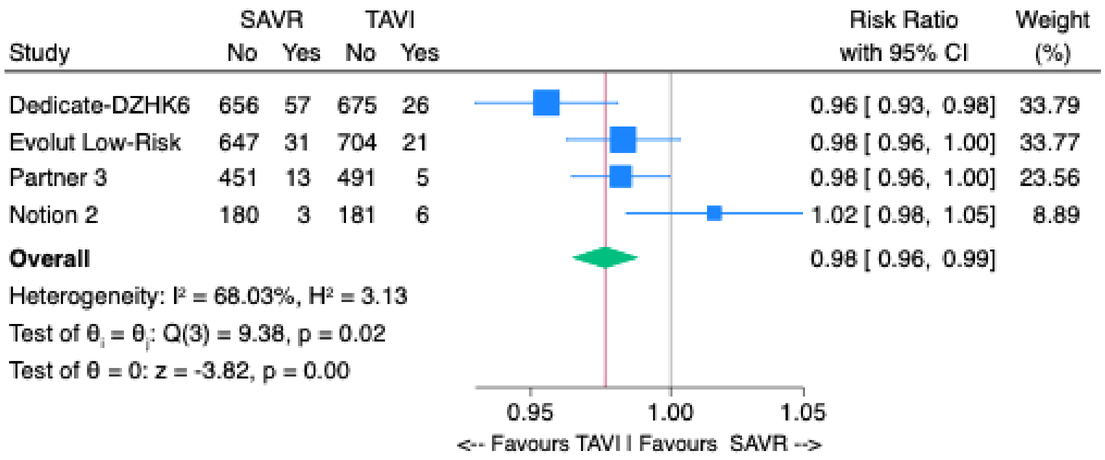

Fixed-effects Mantel–Haenszel model

Death

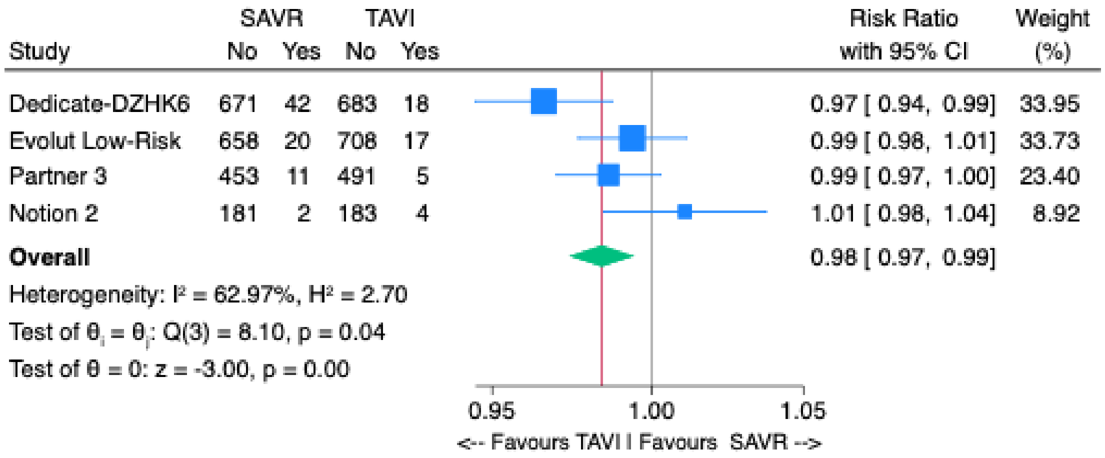

Fixed-effects Mantel–Haenszel model

Disabling Stroke

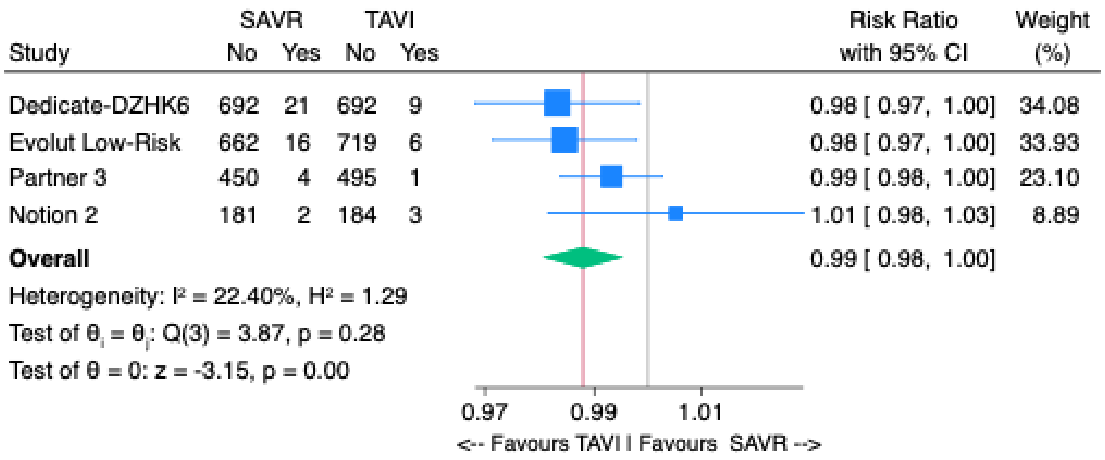

Fixed-effects Mantel–Haenszel model
